# Resistance of Renal Cell Carcinoma to Sorafenib Is Mediated by Potentially Reversible Gene Expression

**DOI:** 10.1371/journal.pone.0019144

**Published:** 2011-04-29

**Authors:** Liang Zhang, Manoj Bhasin, Rachel Schor-Bardach, Xiaoen Wang, Michael P. Collins, David Panka, Prabhakar Putheti, Sabina Signoretti, David C. Alsop, Towia Libermann, Michael B. Atkins, James W. Mier, S. Nahum Goldberg, Rupal S. Bhatt

**Affiliations:** 1 Division of Hematology-Oncology, Beth Israel Deaconess Medical Center, Boston, Massachusetts, United States of America; 2 Division of Interdisciplinary Medicine and Biotechnology, and Genomics and Proteomics Center, Beth Israel Deaconess Medical Center, Boston, Massachusetts, United States of America; 3 Department of Radiology, Beth Israel Deaconess Medical Center, Boston, Massachusetts, United States of America; 4 Department of Radiology, Hadassah Hebrew University Medical Center, Jerusalem, Israel; 5 Department of Pathology, Brigham and Women's Hospital, Boston, Massachusetts, United States of America; 6 Departments of Surgery and Medicine, The Transplant Institute at Beth Israel Deaconess Medical Center, Boston, Massachusetts, United States of America; 7 Department of Medical Oncology, Dana-Farber Cancer Institute, Boston, Massachusetts, United States of America; 8 Kidney Cancer Program of the Dana Farber/Harvard Cancer Center, Boston, Massachusetts, United States of America; Faculdade de Medicina, Universidade de São Paulo, Brazil

## Abstract

**Purpose:**

Resistance to antiangiogenic therapy is an important clinical problem. We examined whether resistance occurs at least in part via reversible, physiologic changes in the tumor, or results solely from stable genetic changes in resistant tumor cells.

**Experimental Design:**

Mice bearing two human RCC xenografts were treated with sorafenib until they acquired resistance. Resistant 786-O cells were harvested and reimplanted into naïve mice. Mice bearing resistant A498 cells were subjected to a 1 week treatment break. Sorafenib was then again administered to both sets of mice. Tumor growth patterns, gene expression, viability, blood vessel density, and perfusion were serially assessed in treated vs control mice.

**Results:**

Despite prior resistance, reimplanted 786-O tumors maintained their ability to stabilize on sorafenib in sequential reimplantation steps. A transcriptome profile of the tumors revealed that the gene expression profile of tumors upon reimplantation reapproximated that of the untreated tumors and was distinct from tumors exhibiting resistance to sorafenib. In A498 tumors, revascularization was noted with resistance and cessation of sorafenib therapy and tumor perfusion was reduced and tumor cell necrosis enhanced with re-exposure to sorafenib.

**Conclusions:**

In two RCC cell lines, resistance to sorafenib appears to be reversible. These results support the hypothesis that resistance to VEGF pathway therapy is not solely the result of a permanent genetic change in the tumor or selection of resistant clones, but rather is due to a great extent to reversible changes that likely occur in the tumor and/or its microenvironment.

## Introduction

A major advance in the treatment of renal cell carcinoma (RCC) over the last few years has been the introduction into clinical practice of antitumor agents that function primarily as inhibitors of vascular endothelial growth factor (VEGF)-driven angiogenesis. The prospect that VEGF receptor (VEGFR) antagonists might be particularly useful in the treatment of RCC – especially the clear cell variant - is predicted from the genetic alterations peculiar to the disease. Approximately 60% of clear cell RCC lack a functional von Hippel-Lindau (VHL) gene as a result of biallelic loss, mutation or hypermethylation [1]. The VHL gene encodes an E3 ubiquitin ligase involved in the oxygen-dependent ubiquitination and proteasomal degradation of HIF-1α and HIF-2α, subunits of transcriptional factors involved in the expression of VEGF and other hypoxia-driven genes. The loss of VHL results in the accumulation of HIF (even in normoxic conditions) and the overproduction of VEGF and various other factors. This feature of clear cell RCC is thought to account for the initial sensitivity of these tumors to VEGF pathway antagonists.

Sorafenib is a multi-tyrosine kinase inhibitor (TKI) whose targets include VEGFR2 and its activity is thought to be based on its action on this target. Sorafenib administration significantly prolonged median progression free survival (PFS) from 2.8 to 5.5 months in a randomized, placebo-controlled phase III trial involving cytokine refractory patients with advanced RCC [2]. Based on these data, sorafenib received FDA approval in late 2005. Subsequently the VEGF pathway blockers sunitinib, bevacizumab and pazopanib were also shown to have sufficient benefit in patients with advanced RCC to merit FDA approval [3].

Although these results are encouraging, there are few if any complete or durable responses to either sorafenib or the other VEGF pathway blockers. Typically tumors develop resistance to sorafenib within a median of 5–9 months, at which point tumor growth resumes even with the continued administration of the drug. While a recent randomized placebo controlled phase III trial reported that the mTOR inhibitor everolimus delayed PFS in patients with sunitinib and/or sorafenib refractory RCC relative to placebo from 1.9 to 4.0 months, there is currently no established consensus for the best treatment approach for patients with RCC that has acquired resistance to sunitinib or sorafenib [4]. In particular, higher response rates and longer PFS have been reported for patients receiving other VEGF pathway blockers, e.g. axitinib after disease progression on sorafenib, or sunitinib after disease progression on bevacizumab [3], [5]–[6].

Resistance could be accompanied by a reversible change in the tumor or could involve a more permanent genetic change in the tumor cells or endothelial cells. While tumors such as lung cancer and chronic myelogenous leukemia develop resistance by a mutation in key signaling pathways in the majority of the malignant cells [7], [8] there is no current available data regarding whether RCC resistance is accompanied by similar tumor cell mutations. Moreover, as the target of sorafenib is most likely the tumor endothelium, it is unlikely that a mutation in this non-malignant population would occur. Furthermore, although the mechanism for acquired resistance to VEGF pathway blockade has yet to be firmly established, the observation that tumors retain their sensitivity to VEGFR inhibitors suggests that permanent changes within the tumor do not universally occur. To address this question, we studied the reversibility of resistance of RCC to sorafenib in two murine human tumor xenograft models.

## Results

### Resistance to sorafenib is reversible

**Figure 1:**
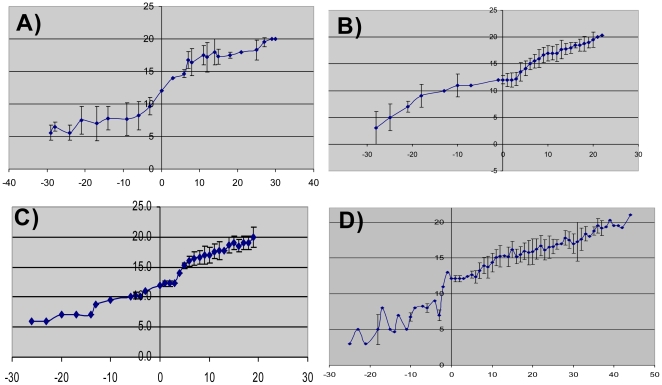
Growth curves of 786-O tumors. Mice with no treatment (A) (n = 9), treated with sorafenib (B) (n = 10), reimplanted into naïve hosts (C) (n = 5) and after a second reimplantation (D) (n = 10). Treatment was started at day 0 and in all tumors treated with sorafenib (treatment naïve (B), and reimplanted resistant tumors (C and D)) there is a period in which the tumors exhibit stabilization of growth (indicated by arrows and quantified in Table 1). X axis represents days on sorafenib and Y axis represents long tumor axis (mm). Average tumor size and standard deviation is shown in growth curves.

We have previously described a model in which the growth of murine RCC xenografts develops resistance to sorafenib as seen in RCC patients. Specifically, we note a period of tumor stabilization after initiation of therapy, defined as time required for the longest tumor diameter to increase by 20% (growth from 12 mm treatment start size to 14 mm) similar to RECIST criteria in patients. This period is followed by a period of more rapid growth similar to the acquired resistance that is seen in patients with RCC [9]. This growth pattern contrasts to the fairly constant growth rate exhibited by untreated tumors. We used this xenograft model to test the reversibility of the resistant phenotype. Untreated tumors (n = 9) grew by 2 mm in 2.6+/−1.2 day while sorafenib treatment (n = 10) led to a 4.9+/−1.5 day period of relative tumor stability (P = 0.0029) (Table 1 and Figure 1 A and B).

**Table 1:** Tumor growth pre and post therapy.

	Time to reach 12 mm(pretreatment growth rate) (days)	Time to grow from 12–14 mm(length of tumor plateau) (days)
**Untreated (n = 9)**	13.8+/−6.6	2.6+/−1.2*
**Sorafenib treated (n = 10)**	15.5+/−6.3	4.9+/−1.5* †
**Sorafenib treatment after reimplantation (n = 5)**	14.0+/−5.0	8.6+/−3.1 †‡
**Sorafenib treatment after second reimplantation (n = 10)**	9.3+/−3.2	9.7+/−3.8‡
	**P>0.09 for all comparisons**	*P = 0.0029, †P = 0.051, ‡ P = 0.56

The time to grow by 2 mm is longer in the initial treated tumors vs. the untreated tumors (P = 0.0029). All comparisons were performed by Student's T test.

We then disaggregated tumors excised at the time of sorafenib resistance and reimplanted the cells into naïve host mice. As shown in Table 1 and Figure 1, the reimplants of previously resistant 786-O cells exhibited a slower growth rate with the initiation of sorafenib treatment (8.6+/−3.1 days period of stabilization), in a manner similar to tumors derived from the sorafenib-responsive parent line (Table 1 and Figure 1C).

Moreover, when the reimplanted tumors that had become resistant a second time were disaggregated and reimplanted into naïve hosts, they maintained their ability to respond to sorafenib a third time with a period of tumor stabilization of 9.7+/−3.8 days (n = 10) (Figure 1D). While the reimplanted tumors seemed to stabilize in response to sorafenib for slightly longer after reimplantation, the tumor growth rate prior to initiation of treatment was similar in the untreated and previously treated xenografts. Specifically, the time to grow from 7 mm to the treatment initiation size of 12 mm was not significantly different among the groups (Table 1). These data support the hypothesis that resistance to sorafenib therapy is at least in part reversible.

### Resistance is accompanied by reversible changes in gene expression

To determine if the changes in gene expression associated with the development of resistance were also reversible and in effort to identify genes that are associated with resistance, total RNA was isolated from untreated tumors harvested at 12 mm, at treatment day 3, treatment resistant (tumors that had grown to 20 mm sacrifice size despite continued treatment), and reimplanted untreated tumors after one and two reimplantations harvested at 12 mm. Gene expression profiling was performed using a comprehensive Affymetrix platform that measures more than 54,000 well-characterized human transcripts and variants, including 38,500 well-characterized human genes. The expression profiling was performed on at least four tumors from each group.

**Figure 2:**
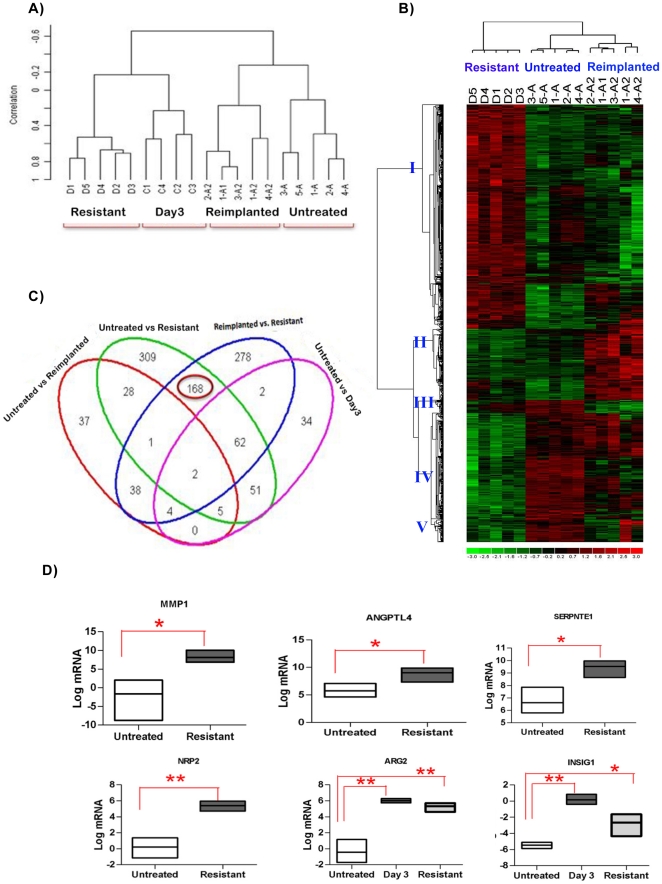
Transcriptional profiling analysis of sorafenib treated tumors. A) Unsupervised Pearson Correlation based cluster of untreated tumors harvested at 12 mm (A), at treatment day 3 (C), treatment resistant (tumors that had grown to 20 mm sacrifice size despite continued treatment) (D), and reimplanted untreated tumors after one and two reimplantations harvested at 12 mm (A1 and A2) after normalizing the data. The treated tumors (D,C) form a separate cluster from control and reimplanted tumors. B) Cologram depicting the different expression patterns of the genes that are either differentially expressed in untreated vs. resistant or reimplanted vs. resistant tumors. The columns represent the samples and rows represent the genes. Gene expression is shown with pseudocolor scale (−3 to 3) with red denoting high expression level and green denoting low expression level of gene. The Genes depict five major expression patterns (marked I to V). C) Venn diagram indicating overlap between differentially expressed genes untreated vs resistant, untreated vs responsive, untreated vs. reimplanted and reimplanted vs. resistant. The differentially expressed genes are extracted using Significantly Analysis of Microarray data (SAM) approach. The green circle shows the 626 transcripts are changed in resistant tumors as compared to untreated tumors. The overlap of the green and pink circles shows the 120 genes that are also differentially expressed at day 3 of therapy. The blue circle shows the 555 transcripts that are differentially expressed in reimplanted as compared to resistant tumors and the overlap of the blue and pink circles show the 70 of the 553 that are altered at day 3 (Responsive). 168 genes are commonly differentially expressed in the untreated and reimplanted tumors as compared to resistant tumors and not changed at day 3 of therapy and are circled. D) Validation of 6 resistance related genes was conducted using untreated, resistant and day3 treatment samples (n = 3 per group). The graphs represent the statistical analyzes of relative mRNA levels after normalization for 18S rRNA levels. The results are expressed using floating bars representing the minimum and maximum values in the group with a line representing the mean. (* P<0.05, ** P<0.001, by Unpaired Student's T test) ANGPTL4, MMP1, SERPINE1 and NRP2 were significantly upregulated at resistance but not at day 3 in the gene expression profiling. ARG2 and INSIG1 were increased at day 3 and then decreased at resistance. The PCR showed results similar to transcriptional profiling.

The heterogeneity in the transcription profile of the tumors was identified by unsupervised clustering reflecting the global similarities between the samples (Figure 2A). The unsupervised clustering was performed using all the transcripts that depict a 1.5 fold change in 20% of the arrays used in the experiment. Unsupervised clustering demonstrated the highest similarity within the biological replicates from each group and the least similarity between the untreated (A, A1, A2) and the treated tumors (C, D). Hierarchical clustering of all samples demonstrated a clear distinction between untreated and on treatment samples. This finding is consistent with the hypothesis that the majority of changes induced in a tumor with sorafenib treatment and, in particular, after the development of sorafenib resistance do not represent permanent changes in the tumors.

The heat map shown in Figure 2B depicts the pattern based comparison of the expression values of 985 unique genes that are significantly differentially expressed in one or more of the following groups: untreated vs. resistant (626), untreated vs. reimplanted (115) or reimplanted vs. resistant tumors (555). The sample wise clustering depicts three different groups corresponding to these biologic traits (horizontal axis). The gene-wise clustering (vertical axis) depicts five major clusters in gene expression profile: Clusters I (505 genes) & IV (235 genes) represent the genes whose expression is altered at resistance and reverts with reimplantation, while clusters II, III and V (161, 29 and 55 genes, respectively) represent differentially expressed genes whose expression does not revert toward baseline with reimplantation. Thus, ∼75% of genes whose expression is altered with resistance appear to revert to baseline with reimplantation.

To better identify the genes that represent the most likely contributors to the reversible component of acquired resistance to sorafenib therapy, we generated a Venn Diagram comparing the various groups (Figure 2C). Also in Figure 2C, we included genes that were differentially expressed at day 3 of sorafenib treatment to identify and eliminate the confounding factor of sorafenib treatment from the subset of differentially expressed genes at resistance that revert with reimplantation. Out of 626 transcripts that differ in resistant tumors as compared untreated controls, 120 were also differentially expressed at day 3 of therapy and thus may represent the effect of sorafenib treatment on the tumors. Similarly out of 555 transcripts significantly modulated in resistant tumors as compared to reimplanted tumors, 70 were also found significantly altered at day 3. All told, 168 transcripts were identified as differentially expressed in resistant tumors as compared to untreated as well as resistant as compared to reimplanted tumors and to not be significantly modulated at day 3. These 168 genes, therefore, represent the most likely contributors to the reversible component of acquired resistance to sorafenib therapy and thus, may represent a “resistance signature”.

To validate the microarrays results, we performed RT-PCR analysis on four genes from the 168 genes that were significantly overexpressed at resistance but not at day 3 (ANGPTL4, MMP1, SERPINE 1 and NRP2); and 2 genes from the 70 genes that were modulated with treatment and resistance (ARG2 and INSIG1). In all cases the relative expression assessed by PCR correlated with the relative expression levels noted in the expression profiling analysis (Figure 2D).

**Figure 3:**
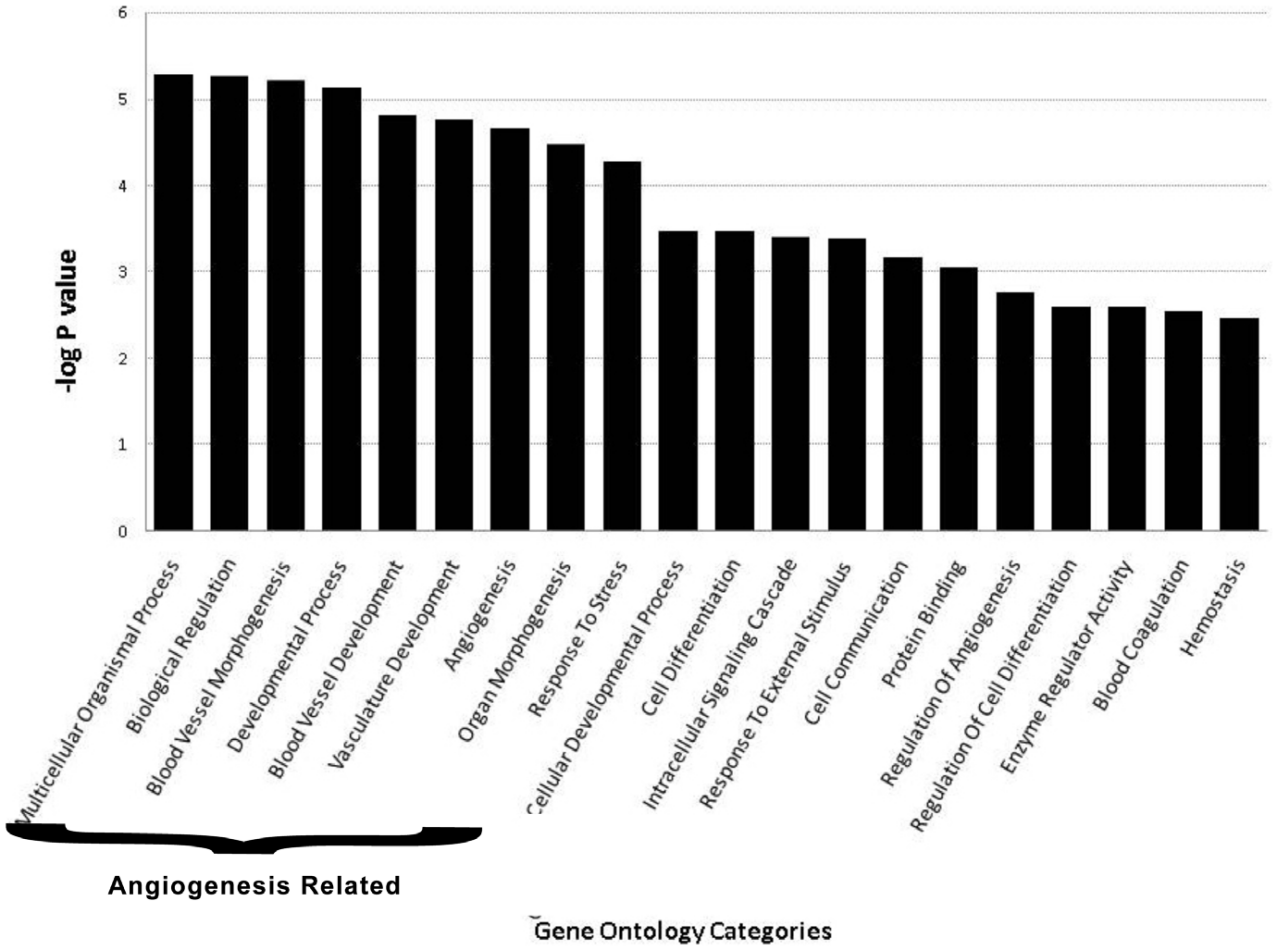
Gene ontology based enrichment analysis. Enrichment analysis of genes that are commonly differentially expressed in untreated vs. resistant and reimplanted vs. resistant tumors. The analysis was performed using DAVID software. The X axis represents the gene ontology categories and Y axis –log of the EASE score. The P value depicts the significance of enrichment, smaller is the P value more significant will be the enrichment. The most highly enriched geneontology categories included blood vessel morphogenesis, angiogenesis and blood vessel/vasculature development.

To gain insights into the broad underlying biology of resistance, gene ontology enrichment analysis was performed on the resistance signature (168 genes) [10]. The top biological processes and metabolic functions that are enriched in the set are shown in Figure 3. All these gene ontology categories are significantly affected (p<0.001). The most highly enriched categories included blood vessel morphogenesis, angiogenesis and blood vessel/vasculature development. The angiogenesis group represents 41 discrete genes. ANGPTL4 and NRP2 are included in the angiogenesis gene ontogeny groups and as mentioned were validated by RT-PCR.

Consistent with gene expression findings, a decrease in CD34 positive blood vessels was noted early in therapy, began to reemerge with resistance [8] and approached the pretreatment level with reimplantation (data not shown).

### Re-exposure to sorafenib leads to a second decrease in tumor blood flow

**Figure 4:**
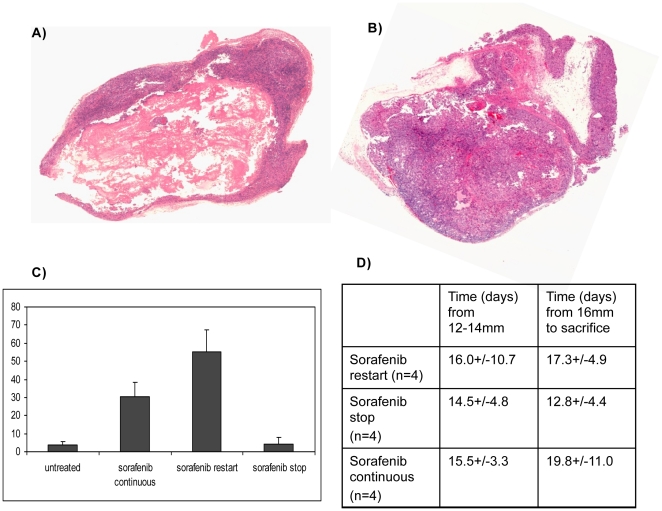
Pathologic analysis of A498 tumors receiving two different regimens of sorafenib. Representative H&E stain showing tumor necrosis in a tumor from a mouse treated with sorafenib at 12 mm and in which sorafenib was stopped when the tumor became resistant (14 mm) and then restarted after the tumor increased from 14 to 16 mm (A: sorafenib restart) and a tumor from a mouse in which sorafenib was started and stopped at the same timepoints but not restarted on therapy at 16 mm (B: sorafenib stop) is shown. Both specimens were harvested when tumors reached 20 mm. The area of necrosis (N) is much more prominent in the tumor in which sorafenib was restarted and this was quantitated and shown in C (N = 3–4 for each arm; P = 0.0027). D shows the timing of growth of the tumors from the 3 arms.

To extend these findings and to confirm our results in a second RCC cell line, mice harboring tumors derived from A498 cells (a VHL deficient RCC cell line) were treated with sorafenib according to 3 schedules. In the first two groups, treatment was initiated at 12 mm and stopped when the tumor long axis reached 14 mm and then either resumed when the tumors increased by another 2 mm or continued to be withheld. In the third group, sorafenib was initiated at 12 mm and not stopped. The time for tumors to grow from 12 -was similar for all treatment arms (n = 12) but the time to grow by another 2 mm from the start of the second treatment was shorter in the arm in which sorafenib was stopped and not restarted (Figure 4D). We did not detect a significant difference in tumor growth rate from the time of sorafenib re-initiation in the mice with discontinuous treatment relative to those who received continuous treatment, (Figure 4D); however, we did note a marked decrease in viable tumor and increase in tumor necrosis with the reintroduction of sorafenib (A) compared to the other two arms (B). As shown in Figure 4C the total area of necrosis of all tumors retreated with sorafenib was 55% and the necrosis in the tumors that were not re-exposed to drug was 4% (n = 4 for each arm, P = 0.0027). Moreover, the percent of necrosis of in the tumors in which sorafenib was stopped was similar to that in the untreated control tumors, while the tumors exposed to continuous sorafenib appeared to exhibit less necrosis than was seen with the reintroduction of sorafenib (30% vs 55% respectively, P = 0.19).

**Figure 5:**
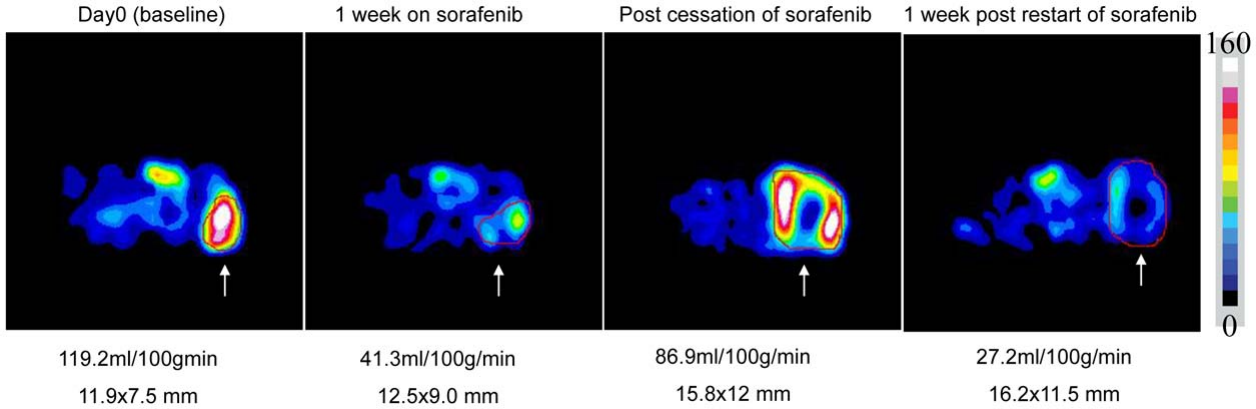
Serial ASL MRI tumor perfusion of A498 derived tumors. Shown are images of an A498 tumor treated with intermittent sorafenib. Tumor perfusion after 1 week of therapy is reduced from 119.2 ml/min/100 g to 41.3 ml/min/100 g. Sorafenib was stopped when the tumor became resistant to sorafenib and tumor perfusion returns after stopping sorafenib (86.9 ml/min/100 g). Sorafenib was restarted after the tumor was allowed to regrow by 2 mm and then, one week after restarting sorafenib, the tumor was devascularized again (27.3 ml/min/100 g). This entire series was duplicated in another mouse and similar relative perfusion was noted, thus a representative series is shown. The tumor is indicated by the region of interest drawn in red and indicated by white arrows.

To further characterize the reversibility of response to sorafenib, serial imaging was performed to assess tumor perfusion during intermittent therapy. As shown in Figure 5, initial treatment with sorafenib led to a reduction in perfusion after 1 week (119.2 decreased to 41.3 ml/min/100 g). When sorafenib was stopped at the time of resistance, tumor perfusion increased (from 38.4 to 86.9 ml/min/100 g); however, 1 week after resumption of sorafenib tumor perfusion was again decreased (from 86.9 to 27.2 ml/min/100 g). This trend was seen in independent imaging series performed in 2 animals receiving the discontinuous treatment schedule. Thus, tumors that have become resistant to sorafenib, after a period off drug, appear to become resensitized to the antiangiogenic effects of the drug and undergo extensive tumor necrosis with reintroduction of therapy.

## Discussion

VEGF pathway blockade is an effective treatment for patients with advanced RCC. However, while the multikinase and VEGFR2 inhibitor sorafenib provides tumor stabilization to a significant proportion of patients, resistance to therapy is inevitable and remains poorly understood. We show evidence in a mouse model that resistance to sorafenib does not appear to result exclusively (or possibly at all) from a stable genetic mutation, as seen with resistance of lung cancer to erlotinib or CML to imatinib [7], [8]. Instead, our results support the hypothesis that acquired resistance to VEGFR antagonists is mechanistically distinct and at least partially mediated by reversible changes in gene expression patterns within the tumor cells and/or microenvironment. In particular, as we show that tumors restore their sensitivity to VEGF blockade after either reimplantation in a naïve host, or a drug holiday, at least a component of the resistance phenotype is reversible. Therefore, if somatic genetic changes occur within the tumor, they too would be responsible for only a component of the resistance phenotype.

Initial experiments used the 786-O line because the relatively short time course in which resistance developed allowed for timely assessment of resistance in multiple rounds of tumor reimplantation. A498 cell line experiments then allowed for confirmation of the concept in another cell line in an experiment that more closely mirrors the clinical situation in which a patient would be exposed to an antiangiogenic therapy and then have treatment held for awhile followed by subsequent treatment with the same or a similar VEGF pathway directed therapy. In this A498 cell line experiments, we were able to demonstrate the clinically relevant end-point of re-induction of tumor necrosis and associated reduction in tumor perfusion on ASL MR imaging with re-exposure to sorafenib.

These findings are consistent with the mechanism of action of sorafenib as an inhibitor of VEGFR2. As the likely target of this agent is the tumor endothelial cell, escape from therapy is likely due to a compensatory change in the tumor cell perhaps triggered by hypoxia, leading to activation of alternative means of supporting angiogenesis. Our biologic data is supported by gene expression data showing that proangiogenic genes are upregulated at time of acquired resistance and largely revert following reimplantion of the resistant tumor into a naïve host. The changes we report are likely tumor cell changes as the Affymetrix chip used was human specific and the cell lines human derived. Thus, while this data does not exclude a significant contribution from mouse stromal cells infiltrating the tumor to the resistance mechanism, it does suggest that any stromal changes may be driven, at least in part, by treatment induced changes in the tumor cells.

A recent study by Tang et al. shows similar biologic effects of sorafenib in mouse models of hepatocellular carcinoma (HCC) [11]. Tang et al found that HCC cell lines that acquired resistance to sorafenib did not exhibit sustained resistance when reimplanted into naïve hosts. Additionally, Hammers et al noted reversal of epithelial to mesenchymal transition and restored sensitivity to sunitinib when a patient derived RCC cell line was implanted into a mouse [12]. The fact that these studies showed similar biologic effects, suggests that the reversibility of resistance observed with our two RCC cell lines, may be a more generalizable phenomenon.

We and others have explored specific mechanisms by which tumors escape VEGFR blockade and have attempted to implicate specific genes whose functional inhibition could prevent of delay the development of resistance phenotype. For example, we have identified in our transcriptome analysis that interferon gamma regulated genes are down-modulated with resistance and have reported that the administration of angiostatic chemokines such as CXCL9 can delay resistance [13]. In addition, we have seen upregulation of the expression of sphingosine kinase, an angiogenic sphingolipid, with resistance and have reported that disruption of this pathway can slow tumor growth [14]. Additionally, others have shown a role for IL-8 upregulation in RCC resistance while FGF, PlGF and c-met have been reported to contribute to VEGF resistance in other tumor models [15]–[18]. Taken together, this work indicates that resistance is likely to be complex and multifactorial and that different factors may play a role in different tumor types or even with different therapies in a single tumor type. Nonetheless, because of the clinical importance of VEGF pathway resistance, continued efforts to identify potentially dominant factors involved in such resistance are clearly warranted.

These data raise several hypotheses for further investigation. For example, if continued sorafenib exposure leads to a reversible form of resistance, is it possible that an intermittent administration schedule might extend the effectiveness of this agent? Additionally, this data suggests that it might be reasonable to “rechallenge” patients with sorafenib or another VEGF pathway inhibitor following a defined treatment break. These hypotheses are supported by clinical observations of antitumor activity seen with 1) sequential administration of VEGF pathway blockers [5], [19] 2) rechallenge with sunitinib after a drug holiday [20], 3) evidence from the recently reported EFFECT trial that intermittent sunitinib therapy produces superior efficacy than continuous therapy [21]. Thus, it is likely that a period off treatment can allow a tumor to reestablish sensitivity to VEGFR TKI therapy, but precise dosing schedules at present remain speculative.

While the tumor regains its ability to respond to sorafenib, our data suggest that some tumor changes in response to sorafenib are not reversible. Such changes could involve the ability of tumors to tolerate the angiogenic blockade by modulating their metabolic needs. While 75% of genes increased in expression with resistance revert when the tumor is reimplanted into a naïve host, the remaining genes whose expression remains perturbed may be of particular interest. While it is possible that some of these persistent changes may be the result of treatment derived mutations within the tumor cells, the fact that they do not block the restoration of treatment responsive lessens their importance. Nonetheless, given that there is current evidence in the literature that antiangiogenic therapy leads to development of more aggressive tumors [22], [23] it is conceivable that some of these processes may contribute to this altered tumor biology. While our data show that the ability of sorafenib to decrease tumor blood flow is reversible, we are in the process of evaluating other potential effects of therapy that may not be reversible with reintroduction of antiangiogenic therapy.

The inability to sustain the initial tumor stabilization or regression induced by VEGF pathway blockers is arguably the most vexing problem now encountered by oncologists who care for patients with RCC. This study provides information regarding the biologic and molecular mechanisms that curtail the initial effectiveness of VEGFR blockers. We are hopeful that better understanding of the mechanisms underlying resistance to VEGFR blockade will enable the creation of treatment schedules and combination regimens that extend the usefulness of VEGFR TKIs in patients with RCC and possibly other malignancies that exhibit sensitivity to anti-angiogenic therapy.

## Materials and Methods

### Cell Culture

A498 and 786-O, two VHL deficient human RCC cell lines[20], were obtained from the American Type Culture Collection (ATCC, Manassas, VA) and cultured for less than one month with aliquots then frozen. Fresh frozen aliquots were used for each experiment. A498 was grown in Eagle's Minimum Essential Medium (EMEM). 786-O cells were cultured in RPMI 1640 medium (Cellgro). All media was supplemented with 2 mM L-glutamine, 10% fetal calf serum and 1% streptomycin (50 µg/ml) and cells were cultured at 37°C with 5% CO_2_.

### Tumor xenograft induction

For subcutaneous xenograft tumor models, female athymic nude/beige mice (Charles River Laboratories, MA) were used. All experiments were approved by the Institutional Animal Care and Use Committee at Beth Israel Deaconess Medical Center. The mice were housed and maintained in laminar flow cabinets under specific pathogen-free conditions and throughout the entirety of the study, all efforts were made to minimize suffering.

Renal cancer cells (786-O or A498) were harvested from subconfluent cultures by a brief exposure to 0.25% trypsin and 0.02% EDTA. Trypsinization was stopped with medium containing 10% FBS, and the cells were washed once in serum-free medium and resuspended in PBS (phosphate buffered saline) as vehicle. Only suspensions consisting of single cells with greater than 90% viability were used for the injections.

To establish RCC tumor xenografts, 786-O or A498 tumor cells were injected subcutaneously (1×10^7^ cells) into the flanks of 6–8 week old mice that were of 20 gm average body weight. Tumors developed in >80% of mice and were usually visible within a few days of implantation. Once they reached a diameter of 3–5 mm, tumors were measured daily with calipers to ensure a consistent size at the outset of treatment. Sorafenib (80 mg/kg, Bayer) was administered 6 out of 7 days per week by gavage beginning when the tumors had grown to a diameter of 12 mm as previously described [9], [25]. Treated and control tumors were again measured daily during sorafenib therapy. Tumor long axis was measured and followed to determine growth curves. 12 mm was used a the prespecificed pretreatment start size in part because this size of tumor size may be sufficiently large to be comparable to a lesion in human clinical setting, but not too large to prevent a period of several weeks before the the mice would need to be sacrificed. It is also an optimal size for tumor perfusion imaging, which is not easy to perform on very small tumors. Growth by 2 mm from that size is the minimal reproducible growth that can be accurately measured by our calipers. We sought to define the relative response as the smallest measurable increase in tumor size. This is the period we have associated with disease stability, similar to that which is seen in patients as it is roughly equivalent to the increase in tumor size (20% increase in long axis by RECIST criteria) that would classify a patient as having progressive disease (and therefore, treatment resistance) while receiving such therapy. Treatment was continued until tumors grew to 20 mm (i.e. the maximum allowable growth by IACUC) at which point the mice were sacrificed. Tumor tissue was obtained pre-treatment, during stabilization/response and at time of resistance for various analyses described below.

### Tumor reimplantation

786-O tumors that had grown to 20 mm on sorafenib treatment were harvested in sterile fashion and prepared for reimplantation. Within 15 minutes of its dissection and removal, the tumor was homogenized with a tissue homogenizer (PowerGen Model 125; Fisher Scientific, Waltham, MA) using aseptic technique, and the tumor cells were washed three times and suspended in sterile PBS (Mediatech, Inc. Herndon, VA.) for a total volume of 0.4 ml per injection. The tumor cell suspension was again injected slowly via a 20-gauge needle subcutaneously into the left flank of mice (1^st^ re-implantation group). Animals were monitored and treated as previously described.

For the second cycle of re-implantation, a reimplanted tumor that had received a second treatment regimen with sorafenib and again reached 20 mm was used as a parent tumor for injections to a third group of naïve hosts (2^nd^ reimplantation). Again, the mice were treated as above the tumors monitored and harvested when they grew to 20 mm.

### Tumor Rechallenge

Mice bearing A498 tumors began sorafenib treatment when tumors reached 12 mm in diameter. Treatment was stopped in mice when tumors reached 14 mm in diameter and then restarted in half of the mice after one week (the average time to increase from 14-16 mm). Perfusion imaging was performed in both groups after 1 week of treatment and mice were sacrificed and tumors harvested for histologic review when the tumors reached 20 mm. Tumors from rechallenged mice were compared to those from mice either remaining off treatment, on continuous sorafenib or untreated controls.

### RNA extraction

RNA was prepared from frozen 786-O tumors using the Qiagen RNA extraction kit (Valencia, CA).

### Gene Expression Analysis

The transcriptional profile of the excised tumors was characterized by oligonucleotide microarray analysis using the human U133A plus 2.0 Affymetrix GeneChip, according to previously described protocols for total RNA extraction and purification, cDNA synthesis, in vitro transcription for production of biotin-labeled cRNA, hybridization and scanning of image output files [26]. The quality of the chip was determined using the affyQCReport package of Bioconductor [27]. The high quality arrays were identified on the basis of the scaling factor, average background, percent present calls and 3′/5′ RNA ratio for normalization. The normalization of data was performed using RMA algorithm in the bioconductor package of R language that consists of background correction, normalization and summarization of the signal values [28], [29].

A hierarchical clustering technique was used to construct an Unweighted Pair Group Method with Arithmetic-mean (UPGMA) tree using Pearson's correlation as the metric of similarity [30]. The expression data matrix was row-normalized for each gene prior to the application of average linkage clustering.

### Preprocessing and identification of differentially Expressed genes

The data were filtered by removing all probes that were absent in all groups of samples e.g. untreated, day 3, resistant and reimplanted. The Absent/Present/Marginal calls for the transcripts were obtained using MAS5 algorithm [31]. The list of the differentially expressed genes between any two groups (e.g. untreated vs. day 3, day 3 vs. resistant) was obtained using the SAM analysis, an implementation of BRB array tool [32]. The class comparison was performed with 100 random permutations, a confidence level of false-discovery rate assessment of 90% and a maximum allowed proportion of false-positive genes of 5% [33]. All these genes have a low likelihood of being false positives. The list of the genes yielded by SAM analysis was further refined using a fold change cutoff of >2 between the control and experimental group to create final lists of differentially expressed genes.

### Geneontology analysis

The Database for Annotation, Visualization and Integrated Discovery (DAVID) was used to identify over-represented gene ontology categories form the significantly differentially expressed genes identified in previous analysis [10]. DAVID is an online implementation of EASE software that produces the list of over-represented categories using jackknife iterative resampling of the Fisher exact probabilities with Bonferroni multiple testing correction. The EASE score is a significance level with smaller EASE scores indicating increasing confidence in overrepresentation. We picked GO categories that have EASE scores of 0.05 or lower as significantly over-represented.

### Complimentary DNA synthesis

Total RNA was converted into cDNA using TaqMan Reverse Transcription kit (Applied Biosystems Inc., Foster City, CA, USA).

### Quantitative real-time PCR analysis

Quantitative real-time PCR (qt-RT-PCR) analysis was performed by a two-step process, a 15-cycle preamplification step (AmpliTaq® DNA Polymerase Kit; Applied Biosystems Inc., Foster City, CA, USA) followed by measurement of mRNA with an ABI PRISM 7900HT Sequence Detection System. For the measurement of mRNA levels of 3 genes (angiopoietin like protein 4 (ANGPTL4), matrix metalloprotease 1 (MMP1), serine protease inhibitor protein 1 (SERPINE1), primers were custom designed and ordered from IDT (San Diego, CA. The sequences for these primers are provided in Table S1. For the measurement of mRNA levels of three genes (neuropilin 2 (NRP2), arginase 2 (ARG2), insulin-induced gene 1 (INSIG1), the TaqMan probe-primer sets were commercially purchased (Assay-on-demand, Applied Biosystems, Inc., Foster City, CA, USA). 2x QuantiFast Probe- or SYBR Green- PCR Master Mix for qt-RT-PCR was purchased from Qiagen (Valencia, CA, USA). These genes were chosen by their expression level in the array and the robustness of their differences in the studies conditions. Amplification was carried out in a total volume of 20 µl for 40 cycles of 3 seconds at 95°C, 30 seconds at 60°C; initial enzyme activation was performed for 3 min at 95°C. For normalizing target gene expression, 18s rRNA (house keeping gene (HKG)) expression was used. The expression measurements were performed on the RNA extracted from untreated, day3 and resistant tumors in duplicate. The results are shown as ratio of target mRNA copy number to 18s rRNA copy number.

### Necrosis Assessment

Hematoxylin and Eosin (H&E) stain was performed on formalin fixed, paraffin embedded tumors and necrosis was assessed. To quantitate total necrotic area, slides were scanned using the Scanscope XT (Aperio Technologies Inc., Visa, CA). For each xenograft tumor, total tumor area as well as areas of necrosis within the tumor were selected and measured using the ImageScope Software (Aperio Technologies Inc).

### Tumor perfusion imaging

Tumor perfusion imaging (Arterial Spin Labeled [ASL] MRI) was performed as previously described [9]. Briefly mice were anaesthetized, and placed in the supine position on a 3 cm in diameter custom-built surface coil. Adhesive tape was used to limit movement. Images were acquired using a 3.0 T whole-body clinical MRI scanner (3T HD; GE Healthcare Technologies, Waukesha, WI). A single slice ASL image was obtained with a single-short fast spin echo sequence (SSFSE) using a background-suppressed, flow-sensitive alternating inversion-recovery strategy. Twenty-four label and control pair images were acquired and averaged for the ASL acquisition. A reference proton density image was acquired by turning off all background suppression and labeling pulses in the ASL preparation. T1 measurement was performed after ASL imaging by using the same imaging sequence at same slice location but with inversion recovery at different inversion times. The single transversal slice of ASL was carefully positioned at the center of tumor, which was marked on the skin with a permanent marker pen for follow-up MRI studies. ASL sequence raw data were saved and transferred to the analysis workstation for image reconstruction by using custom software written within the Interactive Data Language (IDL; research Systems, Boulder, Co). The ASL difference image, between average label and control images, was then converted to quantitative tumor perfusion as previously described [34].

Perfusion was calculated on a pixel-by-pixel basis, and quantitative maps were produced. The quantitative maps and the corresponding proton density reference images were then analyzed by using Image J software (Image Processing and Analysis in Java; National Institutes of Health, Bethesda, MD). To determine tumor perfusion, a region of interest was drawn freehand around the peripheral margin of the tumor by using an electronic cursor on the reference image that was then copied to the perfusion image. The mean blood flow for the tumor tissue within the region of interest was derived, and image window and level were fixed. A 16-color table was applied in 10 mL/100 g/min increments ranging from 0 to 160 mL/100 g/min, with flow values represented as varying shades of black, blue, green, yellow, red, and purple, in order of increasing perfusion.

## Supporting Information

Table S1 Primer Sequences.
(DOC)
