# Supplementary material for: Resistance of Renal Cell Carcinoma to Sorafenib Is Mediated by Potentially Reversible Gene Expression
Source: PLoS One. 2011 Apr 29;6(4):e19144. doi: 10.1371/journal.pone.0019144 (PMC3084751; doi:10.1371/journal.pone.0019144)
Supplement: Table S1 — Primer Sequences. (DOC) [file pone.0019144.s001.doc]

Table S1: Primer Sequences

| MMP-1 F | CATGCGCACAAATCCCTTCT |
| --- | --- |
| MMP-1 R | CATCTCTGTCGGCAAATTCGT |
| SERPINE1-F | ATTCAAGCAGCTATGGGATTCAA |
| SERPINE1-R | CTGGACGAAGATCGCGTCTG |
| ANGPTL4-F | TGGACCACAAGCACCTAGAC |
| ANGPTL4-R | AGTTCACCAAAAATGGCGGAG |
